# Kissinger Method in Kinetics of Materials: Things to Beware and Be Aware of

**DOI:** 10.3390/molecules25122813

**Published:** 2020-06-18

**Authors:** Sergey Vyazovkin

**Affiliations:** Department of Chemistry, University of Alabama at Birmingham, 901 S. 14th Street, Birmingham, AL 35294, USA; vyazovkin@uab.edu

**Keywords:** crosslinking polymerization (curing), decomposition, degradation, liquid and solid state, phase transitions, thermal analysis

## Abstract

The Kissinger method is an overwhelmingly popular way of estimating the activation energy of thermally stimulated processes studied by differential scanning calorimetry (DSC), differential thermal analysis (DTA), and derivative thermogravimetry (DTG). The simplicity of its use is offset considerably by the number of problems that result from underlying assumptions. The assumption of a first-order reaction introduces a certain evaluation error that may become very large when applying temperature programs other than linear heating. The assumption of heating is embedded in the final equation that makes the method inapplicable to any data obtained on cooling. The method yields a single activation energy in agreement with the assumption of single-step kinetics that creates a problem with the majority of applications. This is illustrated by applying the Kissinger method to some chemical reactions, crystallization, glass transition, and melting. In the cases when the isoconversional activation energy varies significantly, the Kissinger plots tend to be almost perfectly linear that means the method fails to detect the inherent complexity of the processes. It is stressed that the Kissinger method is never the best choice when one is looking for insights into the processes kinetics. Comparably simple isoconversional methods offer an insightful alternative.

## 1. Introduction

The thermal behavior of materials is explored broadly by the techniques of differential scanning calorimetry (DSC), differential thermal analysis (DTA), and thermogravimetry (TGA). Kinetic analysis of the data obtained by these techniques provides important insights into the fundamental issues of the reactivity and stability of materials. When it comes to kinetics, the Kissinger method is by far the most popular way of evaluating the activation energy of thermally stimulated processes. The method was introduced in two successive publications [1,2] that, according to the Scopus database, have been cited over 12,000 times. Yet, science is not a popularity contest, so that a routine followed by the majority is not guaranteed to yield the best or even simply a correct result. As a matter of fact, the method is not among the techniques recommended [3] for advanced kinetic studies. Most of the time, it is employed in materials characterization work that among other quantities reports a number that presumably characterizes an energy barrier to a thermal process under study. In this situation, the Kissinger method provides an unbeatably simple way of estimating the activation energy.

While a desired trait, simplicity may carry the risk of trivializing the problem. This applies fully to the Kissinger method as its formalism largely oversimplifies the kinetics of the processes it treats. Nowadays, as never before, the kinetics community concerned with thermally stimulated processes has come to realize that these processes are commonly multi-step [3]. As such, they have more than one energy barrier that controls them, so that the temperature dependence of their rates cannot be described by a single activation energy. In contrast, the Kissinger method yields a single value of the activation energy regardless of the process complexity. In other words, this method is destined to generally miss the actual kinetic complexity. Clearly, this is an essential limitation of the method. Nonetheless, it is just as clear that exposing this limitation is unlikely to stop the enormous usage of the Kissinger method. Respectively, this is not an objective of the present work. Rather, it aims at helping those who use the method to do it in a conscientious manner. It means to use the method with the clear realization that its application does not usually provide adequate insights into the processes kinetics and typically yields the results of a very limited value.

To accomplish the task, we consider a number of the applications of the Kissinger method that include the most popular ones such as chemical reactions, crystallization and glass transition. For each of the processes considered we give a brief theoretical discussion to explain the origins of the complex temperature dependence of the respective rate. Then we apply the Kissinger method to experimental data to see whether the said complexity manifests itself in the application. In addition to that, we briefly discuss some general limitations that are associated with the underlying assumptions of the method.

## 2. Basics of the Method

The simplest form of the Kissinger equation for estimating the activation energy, *E*, is as follows:

(1) $$E=-R\frac{d\ln\left(\frac{\beta }{T_{p}^{2}}\right)}{dT_{p}^{-1}}$$

where *R* is the gas constant, *β* is the heating rate and *T_p_* is the temperature that corresponds to the position of the rate peak maximum. Most commonly *T_p_* is determined as the temperature of the peak signal (maximum or minimum) measured by DSC, DTA or derivative thermogravimetry (DTG).

A curious fact about Equation (1) is that it had been proposed in an obscure paper by Bohun [4] a few years before the famous publications [1,2] by Kissinger. A more instructive form of the Kissinger equation is the integral one:

(2) $$\ln\left(\frac{\beta }{T_{p}^{2}}\right)=\ln\left(-\frac{AR}{E}f^{\prime }\left(\alpha_{p}\right)\right)-\frac{E}{RT_{p}}$$

where $f^{\prime }\left(\alpha \right)=\frac{df\left(\alpha \right)}{d\alpha }$. Equation (2) originates from the basic rate equation of a single-step process:

(3) $$\frac{d\alpha }{dt}=A\exp\left(\frac{-E}{RT}\right)f\left(\alpha \right)$$

where *α* is the extent of conversion of the reactant to products, *t* is the time, *A* is the preexponential factor and *f*(*α*) is the reaction model. A list of the models is available elsewhere [5].

Both Equations (1) and (2) suggest that the activation energy can be evaluated as the slope of the Kissinger plot of $\ln\left(\frac{\beta }{T_{p}^{2}}\right)\;\mathrm{vs}.\;T_{p}^{-1}$. However, Equation (2) indicates that for this plot to be linear the intercept should be a constant independent of the heating rate. This condition is not generally satisfied because *α_p_* is known [6] to depend on *β*. To satisfy it, *f*′(*α*) should be independent of α. This is the case of a first order reaction model, *f*(α) = 1 − *α*, for which *f*′(*α*) = −1. However, for the majority of the reaction models *f*′(*α*) depends on *α* and, thus, on *β*. This introduces some inaccuracy in estimating the value of *E*. As shown by Criado and Ortega [7] for a large variety of models the respective error does not exceed 5% as long as *E*/*RT* > 10.

It needs to be stressed that checking for potential systematic variation of *α_p_* with *β* should be taken as a general prerequisite for using the Kissinger method. The existence of such variation can be an indication of the process complexity as discussed by Muravyev et al. [8]. They have also demonstrated that even a moderate change of 0.06 in *α_p_*, caused by a change of *β* from 1 to 10 K min^−1^, can result in a systematic error in *E* as large as 15%.

The error caused by the dependence of *α_p_* on *β* is eliminated in isoconversional methods [5,9,10]. One of them is the Kissinger-Akahira-Sunose method [11]. It employs the same equations as the Kissinger method (i.e., Equations (1) and (2)) but replaces *T_p_* with *T_α_*. The latter is the temperature related to a given conversion at different heating rates. This is a more accurate way of estimating the activation energy. A critical advantage of an isoconversional method over the Kissinger one is that it affords determining the activation energy, *E_α_*, as a function of conversion. A significant systematic variation of *E_α_* with *α* reveals that the process under study involves more than one step. As shown later, this is an essential piece of kinetic information that the Kissinger method tends to miss.

Obviously, the method is not highly accurate but its numerical accuracy is rather tolerable for many practical purposes. Holba and Sestak [12] have additionally questioned the accuracy of the Kissinger method due to not accounting for the thermal inertia component of the heat flow as measured by heat flux DSC (or DTA). Indeed, it is typically assumed that the process rate is directly proportional to the heat flow, *dQ*/*dt*:

(4) $$\frac{d\alpha }{dt}=\frac{1}{Q_{0}}\frac{dQ}{dt}$$

where *Q*_0_ is the total heat released or absorbed during the process. This assumption is due to Borchardt and Daniels [13], whose analysis suggests that the thermal inertia term can be neglected. Not correcting for this term should unavoidably cause some systematic error in the value of the activation energy. This is because the raw (i.e., uncorrected) DSC peaks appear at somewhat higher temperature than they should, and the magnitude of this temperature shift increases with increasing the heating rate. In regard to the Kissinger method, it means that the *T_p_* values determined from uncorrected DSC peaks are shifted to a higher temperature.

The corrected heat flow is obtained via a relatively simple adjustment:

(5) $$\frac{dQ}{dt}=RHF+\tau \frac{d\left(RHF\right)}{dt}$$

where *RHF* is the raw heat flow as measured by DSC and the second addend is the thermal inertia term. This adjustment, however, requires estimating the time constant *τ*. This is done by analyzing the back tail of a DSC peak measured for melting of a pure metal [14]. The value of *τ* is proportional to the total heat capacity of the sample. On the other hand, the temperature correction is proportional to *βτ*. It means that the effect of thermal inertia decreases with using smaller sample masses and slower heating rates. As a rule of thumb, the International Confederation of Thermal Anlaysis and Calrimetry (ICTAC) recommendations [15] suggest that for kinetic studies the product of the mass and heating rate should be kept under 100 mg K min^−1^.

**Figure 1:**
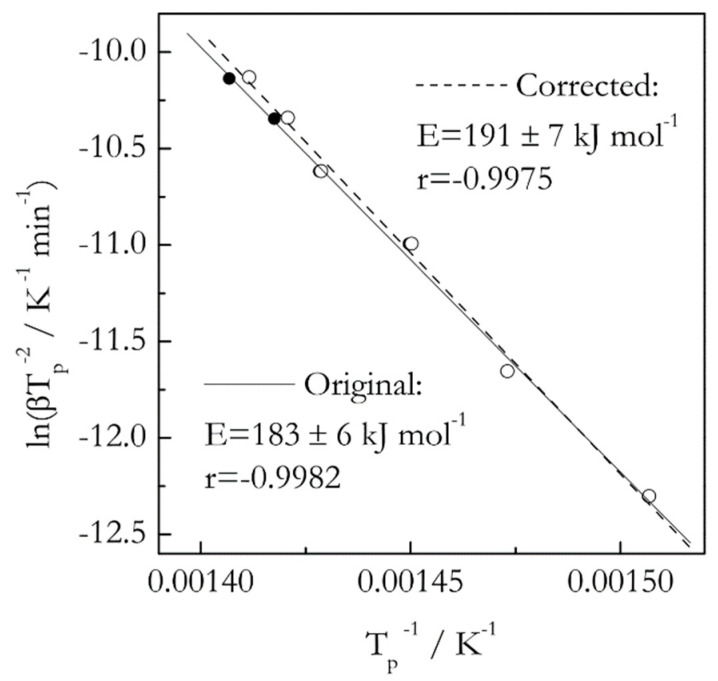
Kissinger plots for thermal degradation of isotactic polystyrene. Data from Liavitskaya and Vyazovkin [18] and Vyazovkin [16]. Solid and open circles represent *T_p_* values obtained respectively from original (raw) data and data corrected for thermal inertia. The difference is obvious only for faster heating rates, 16 and 20 °C min^−1^. Solid and dash lines are the least square fits to original and corrected data.

Our previous study has demonstrated [16] that ignoring thermal inertia in decomposition of 3 mg samples of polystyrene at the heating rates 2–20 °C min^−1^ causes statistically insignificant error when estimating the activation energy by an advanced isoconversional method [17]. Here we use the same data set [18] to compare the effect of thermal inertia on the activation energy estimated by the Kissinger method. The results are presented in Figure 1. As expected, the Kissinger plot for the corrected data is shifted to lower temperatures. The magnitude of the shift is barely detectable at slower heating rates but becomes larger at the faster ones. Upon accounting for thermal inertia, the *E* value has increased from 183 ± 6 to 191 ± 7 kJ mol^−1^, that is, by only 4%. However, with account of the respective uncertainties, the *t*-test suggests that the difference is not statistically significant. This example does not mean that the effect of thermal inertia is negligible in general. Ultimately, the effect is determined by the magnitude of the temperature shift. As long as the latter does not rise above 2–3 °C, the effect should be negligible [16].

Another rarely considered issue related to the accuracy of the Kissinger method is its applicability to the temperature programs other than the one of linear heating. It is noteworthy that the linear heating rate is introduced in the Kissinger derivations by replacing *dT*/*dt* with *β*. However, the related equation is derived for the condition of the rate maximum, that is, the respective values have the meaning of the instantaneous heating rate, *β*_p_. The latter can be defined for a nonlinear heating program that, in principle, affords extending the Kissinger method beyond the standard linear heating [19]. This is important in connection with the sample controlled thermal analysis [20], in which the temperature program is controlled by the response of the reaction rate to heating. This idea is implemented commercially in the techniques of high or maximum resolution TGA. The application of the Kissinger method in the case of nonlinear heating programs has been scrutinized by Sanchez-Jimenez et al. [21]. They have demonstrated that in the case of nonlinear heating the value of *α_p_* can vary significantly with *β*_p_, so that the respective Kissinger plot yields a completely erroneous value of the activation energy. Therefore, the major conclusion here is that before applying the method to the data obtained under a program other than simple linear heating, one needs to make sure that there is no significant variation of *α_p_* with *β*_p_.

In addition, many types of runs are carried out under cooling temperature programs. Whether cooling is linear or not, the data obtained cannot be treated by the Kissinger method. A simple indication is that Equation (2) contains a logarithm of *β*. As already stated, the value of *β* replaces the value of *dT/dt*, so that on cooling *β* is necessarily negative. Obviously one cannot take a logarithm of a negative value, which means that Equation (2) cannot be used for cooling data. Forcing Equation (2) to treat cooling data by dropping the negative sign of *β* results in obtaining completely invalid values of the activation energy [22,23]. That is, the Kissinger method should never be applied to the data obtained on cooling.

## 3. Chemical Reactions

As already mentioned, the Kissinger method is based on Equation (3), which is the rate equation of a single step reaction. The temperature dependence of the latter is determined by a single activation energy that is readily evaluated by the Kissinger method. The problem, though, is that the reactions in the condensed (i.e., solid or liquid) phase typically involve multiple steps and, therefore, face more than a single energy barrier. This has important implications for the temperature dependence of the reaction rate.

Let us consider a very simple reaction that involves two competing steps, each of which follows the same model. The rate of this reaction is:

(6) $$\frac{d\alpha }{dt}=k_{1}\left(T\right)f\left(\alpha \right)+k_{2}\left(T\right)f\left(\alpha \right)\equiv \left[k_{1}\left(T\right)+k_{2}\left(T\right)\right]f\left(\alpha \right)=k_{ef}\left(T\right)f\left(\alpha \right)$$

where the subscripts 1 and 2 designate the rate constants, *k*(*T*), respectively related to the individual steps. In its turn, the temperature dependence of the rate constant is defined via the Arrhenius equation:

(7) $$k\left(T\right)=A\exp\left(\frac{-E}{RT}\right)$$

Experimentally, the activation energy is determined from the slope of the plot of a logarithm of the rate constant vs. reciprocal temperature, that is, as the following derivative:

(8) $$E=-R\frac{d\ln k\left(T\right)}{dT^{-1}}$$

Plugging *k_ef_*(*T*) from Equation (6) into Equation (8) gives:

(9) $$E=\frac{E_{1}k_{1}\left(T\right)+E_{2}k_{2}\left(T\right)}{k_{1}\left(T\right)+k_{2}\left(T\right)}$$

Equation (9) suggests that the experimentally determined activation energy for the above reaction will be temperature dependent, which also means that the respective ln*k*(*T*) vs. *T*^−1^ plot will be nonlinear as illustrated elsewhere [24].

Comparing Equation (8) with Equation (1) suggests that the Kissinger plot for the above reaction would also be nonlinear and the respective activation energy temperature dependent. If this nonlinearity were easy to detect, the application area of the Kissinger method could be extended to multi-step kinetics. In reality, detecting such nonlinearity is not easy and contingent on several conditions. One of them is the number of heating rates used, that is, the number of points on the Kissinger plot. It is nearly impossible to detect the nonlinearity with less than 5 points. However, the typical application of the Kissinger method is limited to 3–4 heating rates. Another important factor, is the width of the temperature range of the *T_p_* values. The wider the range, the better chances to detect the nonlinearity. In experimental terms, a wider range of *T_p_* means a wider range of *β*. The ratio of the maximum to minimum heating rate should be no less than 5. Even if all these conditions are met, the nonlinearity may still escape detecting because the difference in the activation energies of the individual steps is not large enough.

**Figure 2:**
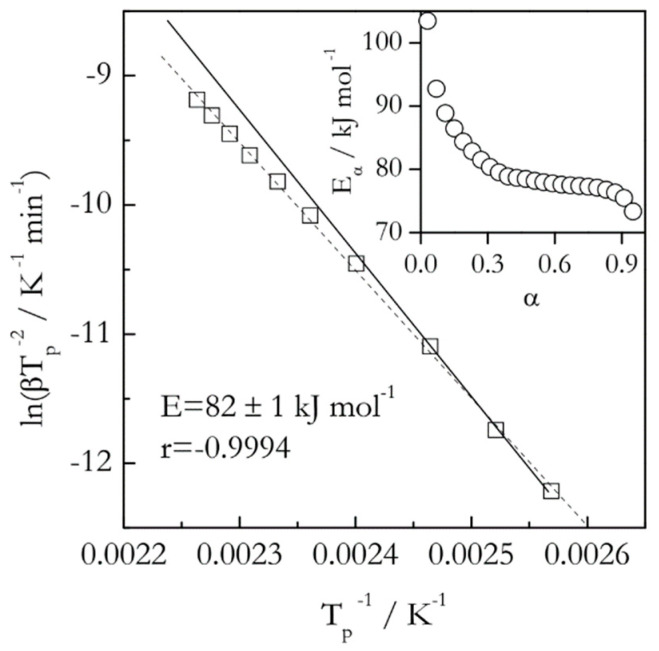
Kissinger plot for thermal dehydration of CaC_2_O_4_ H_2_O. Dash line is least square fit to experimental data (squares). Solid line is a fit to the three lower temperature data points. It demonstrates that the angle of the slope changes for higher temperature data. Inset shows variation in isoconversional activation energy. Data from Liavitskaya and Vyazovkin [25].

A much more sensitive way of detecting the reaction complexity is to use an isoconversional method [5,9,10] that allows one to determine the activation energy as a function of conversion. As an example, Figure 2 provides a comparison of the Kissinger plot with a dependence of the isoconversional activation energy (*E_α_*) on conversion for the thermal decomposition (dehydration) of calcium oxalate monohydrate as measured by DSC [25]. As one can see, the activation energy estimated by an isoconversional method varies from about 105 to 75 kJ mol^−1^. This means that the respective Kissinger plot should be nonlinear. In particular, its lower temperature part should have a steeper slope than the high temperature one. This actually is the case, if one looks very closely. Yet, the change in the angle of the slope is a little over 3° and, thus, is easy to miss. Furthermore, treating this plot as linear, that is, ignoring the small nonlinearity, yields a high value of the correlation coefficient (*r*), which means that statistically this nonlinearity insignificant. All this illustrates how insensitive the Kissinger method is in detecting the reaction complexity. This is especially alarming considering that this Kissinger plot includes 10 heating rates ranging from 0.75 to 20 °C min^−1^ and covering a 53 °C interval.

Naturally, the question can be raised whether the statistically insignificant nonlinearity is important. An answer depends on the purpose of the kinetic study. If one simply needs to obtain a ballpark estimate for the activation energy, such nonlinearity can be ignored. However, it is critically important when one strives for a mechanistic understanding of the estimate. For instance, the decreasing dependence of *E_α_* shown in Figure 2 is not just something encountered in a particular instance of dehydration of calcium oxalate monohydrate. It is a general phenomenon observed for a wide variety of reversible decompositions [26] that include dehydration of diverse crystal hydrates. For this type of processes, the activation energy depends on the equilibrium pressure, *P_0_*, of the gas product as [18]:

(10) $$E=E_{1}+\Delta H^{0}\frac{P}{P_{0}-P}$$

where *E*_1_ is the activation energy of the forward reaction, Δ*H*^0^ is the reaction enthalpy and *P* is the partial pressure of the gas product. According to Equation (10), *E* should decrease with increasing temperature because *P_0_* increases, making the second addend increasingly smaller. Clearly, none of that information can be gained from the single value 82 ± 1 kJ mol^−1^ estimated by the Kissinger method (Figure 2).

It is worth noting that Agresti [27] has proposed a modification to the Kissinger method that accounts for the pressure dependence. As expected, the resulting Kissinger plots are nonlinear and their curvature increases dramatically in the vicinity of equilibrium temperatures.

**Figure 3:**
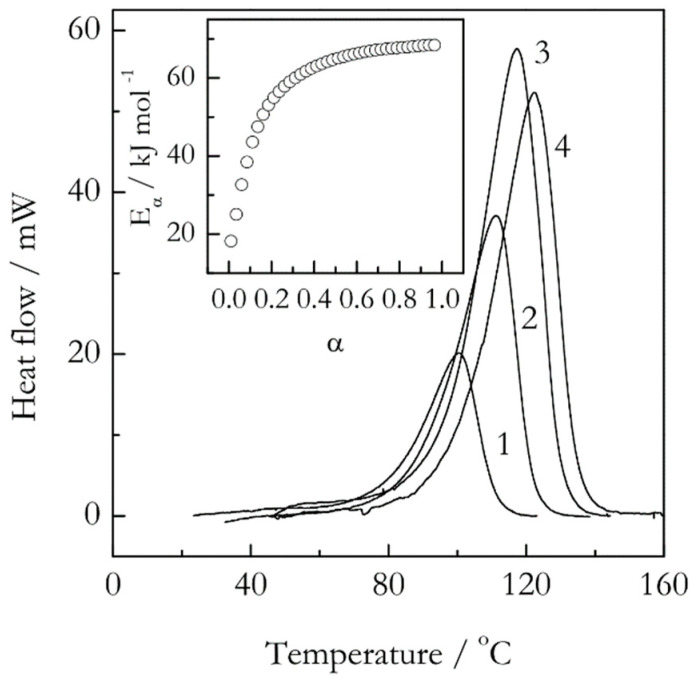
DSC curves for crosslinking polymerization of an epoxy-anhydride system. The numbers by the curves are heating rates in °C min^−1^. The inset shows the conversion dependence of the isoconversional activation energy. Adapted with permission from Vyazovkin and Sbirrazzuoli [29]. Copyright 1999 Wiley-VCH.

Concerning the reaction complexity, there is a common belief that it has to manifest itself via the rate peaks that reveal shoulders or other aberrations of the regular bell-shaped form. Definitely, discovering such features is a sign of the reaction complexity. However, the opposite is not true, meaning that the absence of such features in DSC, DTA or DTG peaks does not mean that the reaction is simple, that is, single step. Figure 3 illustrates such situation in the case of epoxy-anhydride crosslinking polymerization (sometimes termed as curing). It is seen that the respective DSC peaks are of regular bell-shaped form without any obvious aberrations. Yet, the isoconversional activation energy demonstrates a significant increase from about 20 to 70 kJ mol^−1^, which again results from a multi-step reaction mechanism [28] At the same time, the Kissinger plot is almost perfectly linear (*r* = −0.9999) and yields a single value of the activation energy, 71 kJ mol^−1^ [29]. The latter obviously gives no hints regarding the reaction complexity.

Of course, there are cases of single-step reactions. These ordinarily are multi-step reactions whose overall kinetics is limited or dominated by one step. In these cases, the Kissinger method could serve as a basis for an adequate kinetic analysis. The problem, though, is that the method does not typically possess the sufficient sensitivity to differentiate reliably between the single and multi-step kinetics. The occurrence of single-step kinetics can be easily detected by an isoconversional method as the absence of any significant dependence of *E_α_* on *α*. However, employing an isoconversional method for such purpose immediately makes the use of the Kissinger method redundant.

Last but not least, the Kissinger method is a part of the ASTM E698 technique for Arrhenius kinetic constants for thermally unstable materials [30]. This technique relies on using a single value of the activation energy estimated by the Kissinger method to make predictions of a material behavior under isothermal conditions. Unfortunately, in the case of the reaction complexity this technique produces rather poor predictions, that is, significantly less accurate than the ones produced via isoconversional methods that use variable activation energy [31,32].

## 4. Crystallization

There are about as many publications on the application of the Kissinger method to crystallization as to chemical reactions. The applications are so common that sometimes one can see the claims that the method was proposed by Kissinger for estimating the activation energy of crystallization. This, of course, is absolutely false. In reality, neither of his seminal papers [1,2] even contains the word “crystallization.”

To understand the problems with this particular application, one needs to recognize that the temperature dependence of the crystallization rate differs dramatically from that of the reaction rate. Decreasing temperature makes chemical reactions to proceed slower. Crystallization rate depends on supercooling, Δ*T* = *T_m_* − *T* with respect to the equilibrium melting temperature, *T_m_*. At small supercoolings, crystallization accelerates with decreasing temperature until reaching the maximum rate at some temperature *T_max_*. At large supercoolings, that is, at temperatures below *T_max_*, the crystallization rate decreases with decreasing temperature.

This complex temperature dependence cannot be described by a single Arrhenius equation, which is the basis of the Kissinger method. It is described well by the models of nucleation or nuclei growth that combine the Arrhenius kinetics with the underlying thermodynamics of crystallization. The rate of crystallization can be limited by the formation of nuclei or by the growth of existing nuclei. The temperature dependence of the nucleation rate is adequately represented by the Turnbull and Fisher model [33]:

(11) $$n=n_{0}\exp\left(\frac{-E_{D}}{RT}\right)\exp\left(\frac{-\Delta G^{*}}{RT}\right)$$

where *n* is the nucleation rate constant, *n*_0_ the preexponential factor, *E_D_* is the activation energy of diffusion and ΔG^*^ is the free energy barrier to nucleation. The size of this barrier for a spherical nucleus is as follows:

(12) $$\Delta G^{*}=\frac{16\pi \sigma^{3}T_{m}^{2}}{3{\left(\Delta H_{m}\right)}^{2}{\left(\Delta T\right)}^{2}}=\frac{\Omega }{{\left(\Delta T\right)}^{2}}$$

where *σ* is the surface energy (surface tension), Δ*H_m_* is the enthalpy of melting per unit volume and Ω is a constant that collects all parameters that do not practically depend on temperature. The derivations can be found in various sources, for example, References [9,34,35,36,37,38].

If the crystallization rate, *u*, is limited by the growth of existing nuclei, it depends on temperature as follows [38]:

(13) $$u=u_{0}\exp\left(\frac{-E_{D}}{RT}\right)\left[1-\exp\left(\frac{\Delta G}{RT}\right)\right]$$

where *u*_0_ the preexponential factor and Δ*G* is the difference in the free energy of the final (crystalline) and initial (liquid) phase. Surprisingly, the monographic literature does not consider Equation (13) as commonly as Equation (11). Thus, it needs a few comments here. First, it is readily derived as the difference between the rates of the forward and reverse transition. For the forward rate, the energy barrier is *E_D_*, whereas for the reverse rate it is *E_D_* − Δ*G* (Δ*G* < 0). The frequency (preexponential) factor is assumed the same for both rates.

Second, the free energy terms in Equations (11) and (13) differ entirely in their meaning. The ΔG^*^ term in Equation (11) is the energy barrier that makes it a positive value. The Δ*G* term in Equation (13) is the free energy change for a spontaneous process and, thus, negative. Note that Equation (13) is sometimes written with a negative sign in front of Δ*G* when it is referred to as the driving force. The latter in the strict thermodynamics sense [39] should be a positive quantity. This, however, may create extra confusion of dealing with positive Δ*G* for a spontaneous process. One way or another, the argument of the exponential function in the bracketed term of Equation (13) must be negative.

Despite their differences, Equations (11) and (13) suggest the existence of the rate maximum. In both equations, the acceleration at small supercooling is due to the thermodynamic terms. In Equation (11), it occurs because the nucleation barrier ΔG^*^ decreases with increasing the supercooling (Equation (12)), that is, with decreasing temperature. In Equation (13), the dependence on supercooling is introduced via an approximate equality [35,36,38]:

(14) $$\Delta G=\Delta H_{c}\left(\frac{T_{m}-T}{T_{m}}\right)\equiv \Delta H_{m}\left(\frac{T-T_{m}}{T_{m}}\right)$$

where Δ*H_c_* is the enthalpy of crystallization. Then, as temperature lowers and supercooling increases the value of Δ*G* becomes increasingly more negative. As a result, the exponential function in the bracketed term (Equation (13) decreases toward zero, whereas the term itself increases toward unity. Thus, the acceleration is associated with the bracketed term that becomes larger at larger supercoolings.

In both cases (Equations (11) and (13)), the thermodynamic acceleration is counteracted by the diffusional retardation that originates from continuously growing viscosity of the melt. This behavior is represented by the exponential term containing *E_D_*. At some point the diffusional retardation starts to outweigh the thermodynamic acceleration, so that the rate begins to drop with decreasing temperature. As a result, the rate passes through a maximum.

**Figure 4:**
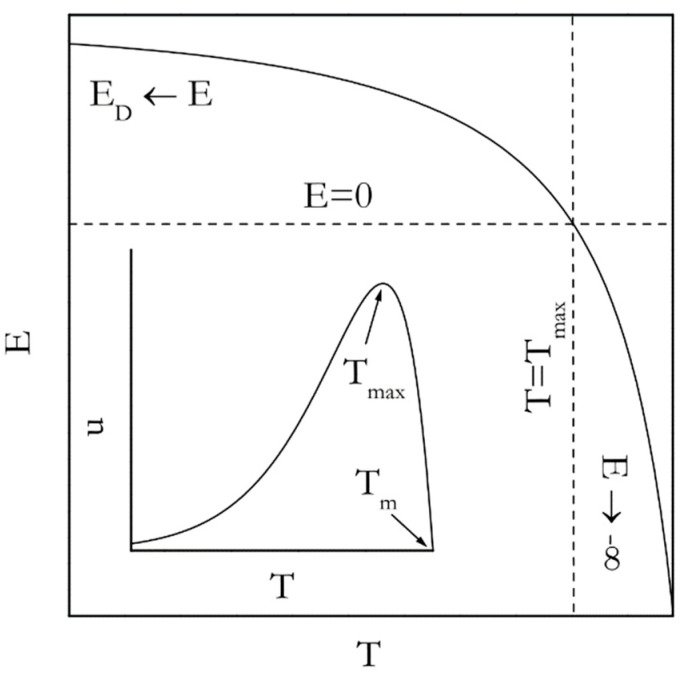
Temperature dependence of the activation energy according to Equation (15). Inset shows temperature dependence of the growth rate according to Equation (13).

Figure 4 (inset) displays the temperature dependence of the rate derived by combining Equations (13) and (14). Such dependence for the nucleation rate (*n* in Equation (11)) can be seen elsewhere [40]. Either of these dependencies passes through a maximum. However, in general the growth process tends to demonstrate the maximum at *T_max_*, which is larger (closer to *T_m_*) than that for the nucleation process.

Most relevant to the Kissinger analysis is that the existence of the rate maximum on the temperature dependence entails the inversion of the sign of the experimentally determined activation energy. This is unavoidable because the activation energy is determined by the sign of the temperature derivative of the rate. In the temperature region above *T_max_* the rate decreases with increasing temperature; the sign of the experimental activation energy is negative. However, below *T_max_* the rate increases as temperature rises so that the sign is positive. An analytic expression for the temperature dependence of the activation energy is arrived at by plugging *u* for *k*(*T*) in Equation (8) that yields the following:

(15) $$E=E_{D}+\frac{\Delta H_{m}\exp\left[\frac{\Delta H_{m}\left(T-T_{m}\right)}{RTT_{m}}\right]}{\exp\left[\frac{\Delta H_{m}\left(T-T_{m}\right)}{RTT_{m}}\right]-1}$$

The resulting dependence is depicted in Figure 4. Indeed, it is seen that *E* takes on large negative values in the vicinity of *T_m_* but increases toward 0 as temperature drops toward *T_max_*. Yet, in the limit of large supercoolings (*T* < *T_max_*) *E* is positive and decreases from *E_D_* toward 0 as temperature rises. The nucleation model (Equation (11)) gives rise to a different analytic expression for the temperature dependence of the activation energy [9], viz.:

(16) $$E=E_{D}-\Omega \left[\frac{2T}{{\left(T_{m}-T\right)}^{3}}-\frac{1}{{\left(T_{m}-T\right)}^{2}}\right]$$

Nevertheless, the dependence predicted by Equation (16) is very similar to the one predicted by Equation (15) and has exactly the same asymptotes, that is, *E_D_* and −∞ for the infinitely large and small supercooling respectively.

The above has direct relevance to the experimental studies of the crystallization kinetics. It needs be recognized that experimentally the temperature region above *T_max_* is accessed by cooling melts, whereas the one below *T_max_* by heating glasses. The applications of the Kissinger method to crystallization of melts are usually encountered in the field of polymers. As explained earlier, the Kissinger method cannot be applied to the data obtained on cooling and when forced to do that yields entirely erroneous values of the activation energy [22,23].

**Figure 5:**
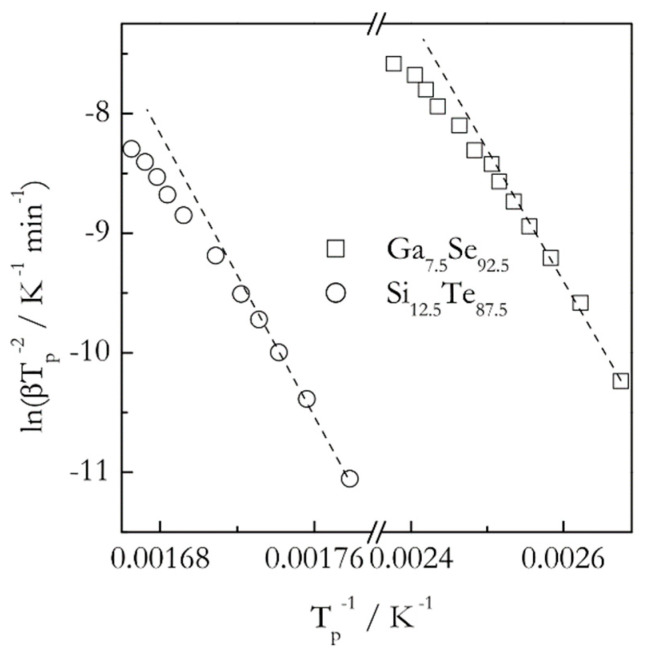
Kissinger plots for crystallization of Ga_7.5_Se_92.5_ (squares) and Si_12.5_Te_87.5_ (circles) glasses. Dash lines connecting three lowest temperature points are a guide for the eye to better visualize the nonlinearity. The heating rates range from 5 to 90 °C min^−1^. Data from Abu El-Oyoun [41,42].

When the Kissinger method is applied to crystallization of glasses, it yields positive activation energies that are commonly reported as constant temperature independent values. On the other hand, the theory predicts the activation energy of the glass crystallization to decrease with increasing temperatures. It means that the corresponding Kissinger plots should be nonlinear, or, more precisely, concave down. As already noted, detecting the curvature requires using multiple heating rates spread over a broad range. For example, the effect is detectable (Figure 5) in the data obtained for crystallization of Ga_7.5_Se_92.5_ and Si_12.5_Te_87.5_ glasses [41,42]. Note that in both cases the range of the heating rates used is unusually broad, 5–90 °C min^−1^. However, if one looks at these plots within a more typical range 5–25 °C min^−1^ (first five points corresponding to the lower temperatures) the curvature is practically unnoticeable. Expectedly, it is much easier to detect a variation in the activation energy by using an isoconversional method. For these two glasses the isoconversional activation energy decreases 1.5 times (from 85 to 55 kJ mol^−1^ for Ga_7.5_Se_92.5_ and from 200 to 130 kJ mol^−1^ for Si_12.5_Te_87.5_) in the temperature range of crystallization [41,42]. It should be emphasized that the curvature of the Kissinger plots for crystallization increases dramatically when using ultrafast scanning calorimetry [43]. This technique permits employing both much faster heating rates and much broader range of them.

## 5. Glass Transition

The glass transition appears to be the third most important application area of the Kissinger method. One of popular models used for describing the glass transition kinetics is the Tool-Narayanaswami-Moynihan model (TNM) [44,45,46]. It can be presented as follows:

(17) $$\ln\tau ={\ln\tau }_{0}+\frac{xE}{RT}+\frac{\left(1-x\right)E}{RT_{f}}$$

where *τ* is the relaxation time, *τ*_0_ is the preexponential factor, *x* is the nonlinearity parameter and *T_f_* is the fictive temperature. The relaxation time in Equation (17) is an analog of the reciprocal rate constant in the Arrhenius equation. The model indicates that the whole process of the glass transition is driven by a single constant activation energy. This is a simplification also known as thermorheological simplicity, which, by no means, is the general rule for the relaxation behavior [47]. An important limitation of the model is that it, in particular, predicts the Arrhenius temperature dependence for viscosity, whereas this dependence is generally of the Williams-Lander-Ferry (WLF) [48] or Vogel-Tammann-Fulcher (VTF) [37,49,50] type.

Based on Equation (17), Moynihan et al. [51] have proposed methods of estimating the activation energy of the glass transition from either cooling or heating data. For heating, *E* is evaluated from the heating rate dependence of the glass transition temperature:

(18) $$E=-R\frac{d\ln\beta }{dT_{g}^{-1}}.$$

In their paper, Moynihan et al. point out that *T_g_* can be defined from calorimetric measurements as temperature of the extrapolated onset or inflection point or the heat capacity maximum. The latter corresponds to the endothermic peak that appears on heating in DSC at the end of the glass transition event.

The appearance of this peak, sometimes referred to as the glass transition peak, has inspired numerous applications of the Kissinger method for determination of the activation energy of the glass transition. Unfortunately, most of these application have been wrong. Apparently, many believe that simply observing a DSC peak that shifts with the heating rate justifies the application of the Kissinger method. This is certainly not true in the case of the glass transition. The problem is not specific to the Kissinger method itself, although it may appear as such [52]. Rather, it arises from heating the glasses not having a proper thermal history. As stressed by Moynihan et al. [51,53,54], obtaining a correct value of *E* from Equation (18) requires creating a glass of a specific thermal history. Namely, immediately before heating the glass has to be cooled at the rate, which is equal (or proportional) to the rate of heating. Also, cooling must occur from the equilibrium state, that is, from well above *T_g_* down to well below *T_g_*. Using other thermal histories gives rise to the *E* values that can deviate dramatically from the correct one [54].

While demonstrated [54] in the case of Equation (18), the importance of using the proper thermal history applies fully to the application of the Kissinger method. As a matter of fact, both the Moynihan (18) and Kissinger (1) equations yield nearly identical values of *E* when *T_g_* is estimated as the peak temperature of the glass transition and they both produce equally wrong values in the case of not using the proper thermal history [55]. Nevertheless, the application of Equation (18) to *T_g_* = *T_p_* when using the proper thermal history does give rise to correct values of *E* [56]. Putting all these results together, we can conclude that one can use the Kissinger method to obtain the correct values of *E* as long as the glass transition measurements are performed on a sample exposed to the proper thermal history, for example, when using heating at *β* immediately preceded by cooling at *−β*, as mentioned before. More importantly, it makes no sense trying to determine the *E* values by the Kissinger or Moynihan method by heating the as-is glass samples. The resulting values would be largely the fortuitous ones that are impossible to interpret in a meaningful way.

Assuming that the glass transition measurements are performed under a proper thermal history, we can now return to aforementioned limitation of the TNM model associated with the oversimplified (i.e., Arrhenius) treatment of the temperature dependence of the relaxation time or viscosity. As stated, the more general is the WLF or the VTF dependence. The VTF equation for the relaxation time is:

(19) $$\ln\tau ={\ln\tau }_{0}+\frac{B}{T-T_{0}}$$

where *B* is a constant and *T*_0_ is a reference temperature. The respective Arrhenius plot, ln*τ* vs. *T*^−1^ is nonlinear and gives rise to the activation energy that decreases with increasing temperature as follows:

(20) $$E=R\frac{BT^{2}}{{\left(T-T_{0}\right)}^{2}}$$

A similar dependence derives [48] from the WLF equation.

This brings about an important question as to whether the activation energy of the glass transition should be a constant or temperature dependent (i.e., variable) value. Based on the Adam-Gibbs theory [57], the activation energy in the Arrhenius equation is proportional to the size of the region that rearranges cooperatively during the transition. This size is inversely proportional to the configurational entropy that increases with *T* so that the experimental activation energy is expected to decrease with *T*. In practice, one may or may not detect this variation depending on the dynamic fragility [58] of the systems studied. According to Angell [58], there are strong and fragile glassformers that demonstrate distinctly different ln*τ* vs. *T*^−1^ plots. For the strong glass formers, these plots are nearly linear, that is, Arrhenian. For the fragile ones, they are nonlinear, that is, of the WLF/VTF type. Typical examples of the strong and fragile glassformers respectively are inorganics and polymers. On the other hand, organics and metals tend to fall between those two limits.

**Figure 6:**
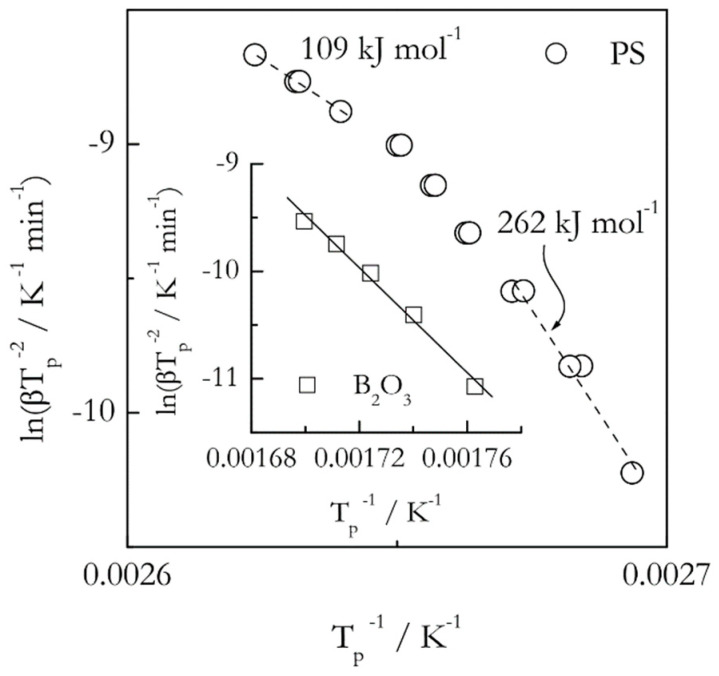
Kissinger plots for the glass transition in polystyrene (PS, circles) and boron oxide (B_2_O_3_, squares). For PS, the plot is visibly nonlinear, the activation energy decreases with temperature, the heating rate range 2.5–25 °C min^−1^. For B_2_O_3_, the plot is practically linear, the activation energy is nearly constant, 203 ± 11 kJ mol^−1^, the heating rate range 5–25 °C min^−1^. Data from Vyazovkin et al. [59,60].

In any event, when it comes to the Kissinger analysis of the glass transition one may obtain either linear or nonlinear Kissinger plots depending on the fragility of the systems studied. Examples of such plots are shown in Figure 6 for two glasses: boron oxide (B_2_O_3_) and polystyrene (PS). The *T_p_* values have been extracted from the previously published DSC data [59,60] As seen from the figure, the Kissinger plot is practically linear for the strong glassformer, boron oxide, whereas it is nonlinear for the fragile one, polystyrene. That is, the activation energy of the glass transition is expected to be practically constant in the former case but temperature dependent in the later one. Note that detecting nonlinearity of the Kissinger plots requires using multiple heating rates in relatively broad range. Much more sensitive way of detecting a variation in the activation energy of the glass transition is to employ an isoconversional method that demonstrates clearly that the variability of the activation energy is proportional to the fragility [61]. Remarkably, the method has been capable of detecting a minor variation in the activation energy even in the case of boron oxide [60].

## 6. Melting and Other Processes

In addition to the three major application areas discussed above, the Kissinger method has been used for treating some other processes. Although these applications are relatively scarce, they are still of research interest. The most common and, perhaps, most confusing is melting. A detailed discussion regarding the theory and practice of the melting kinetics is given elsewhere [40]. Here, we only reiterate a few important points directly relevant to the Kissinger method.

The basic thermodynamics suggests that on heating of a substance its temperature remains constant throughout the melting process. This temperature is the equilibrium melting temperature, which is independent of the heating rate. The confusion may arise from the fact that the position of the DSC peak (i.e., *T_p_*) measured for melting usually demonstrates a noticeable increase with the heating rate. This effect is observed because the DSC peaks are typically presented not as a function of the sample temperature but as a function of the reference (furnace) temperature. The latter obviously increases during continuous heating at the rate *β*. In this situation, the value of *T_p_* represents the reference temperature, at which the substance has finished melting. This temperature shifts with the heating rate according to the following equation [62]:

(21) $$T_{p}-T_{m}=\sqrt{2R_{sf}\Delta H_{m}m\beta }$$

where *R_sf_* is the thermal resistance and *m* is the mass.

As seen from Equation (21), for melting the shift of *T_p_* with *β* is determined by physical parameters other than the activation energy. It means that, as a rule, the application of the Kissinger method to the *T_p_* vs. *β* data would yield a number that does not represent the activation energy of melting. An exception to this rule are the compounds that undergo real superheating, that is, the compounds, whose temperature does rise during melting. For certain reasons [40], melting with superheating cannot be identified by plotting DSC signal against the sample temperature. A simple yet informative test [63] is checking whether the melting peak width increases with increasing either the heating rate or the sample mass. If it does, the melting occurs without superheating and the Kissinger method cannot be applied.

The absence of the aforementioned DSC peak broadening is indicative of melting with superheating. A more definitive (quantitative) criterion is based on determining the value of the exponent *z* in Equation (22):

(22) $$T_{p}=T_{m}+B\beta^{z}$$

where *B* and *z* are fit parameters. According to Toda [63], the *z* values markedly smaller than 0.5 indicate that melting occurs with superheating. In this circumstance, the Kissinger method can be applied to obtain an estimate for the activation energy of melting.

**Figure 7:**
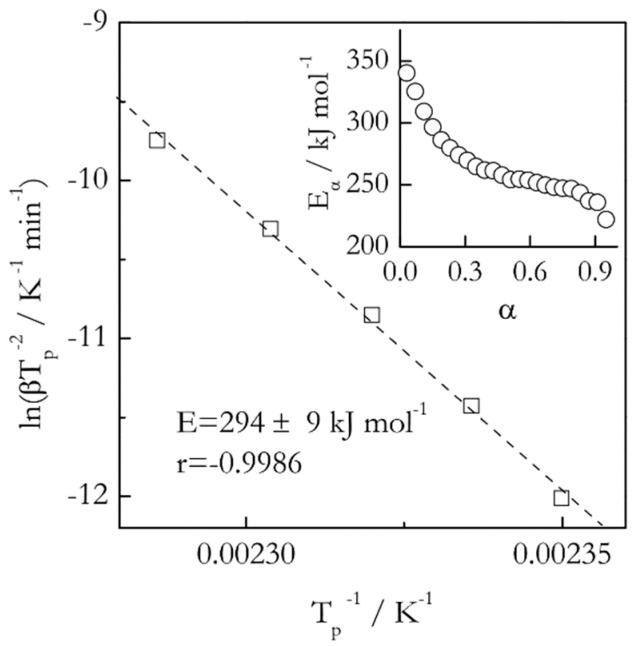
Kissinger plot for melting of glucose. Dash line is least square fit to experimental data (squares). Inset shows variation in isoconversional activation energy (circles). The heating rate range 1–11 °C min^−1^. Data from Liavitskaya et al. [67]. Reproduced from Ref. [67] with permission from the PCCP Owner Societies.

As an example of melting with superheating we consider the case of glucose. Superheating of this substance was reported by Tammann [64] and then reconfirmed by Hellmuth and Wunderlich [65]. For this compound, the *z* value (Equation (22)) has been found [66] to be 0.2 that confirms the occurrence of superheating. Applying the Kissinger method to the DSC data on glucose melting [67] gives rise to a plot (Figure 7) that is practically linear with a significant correlation coefficient (*r* = −0.9986). Correspondingly, the method yields a single constant activation energy 294 kJ mol^−1^.Nonetheless, an isoconversional method yields activation energy that decreases from about 350 to 230 kJ mol^−1^.

Important is that a decrease of the experimental activation energy of melting with increasing temperature is justified theoretically [40]. Theoretically, the kinetics of melting is treated by the nucleation and nuclei growth models (Equations (11) and (13)), that is, just as the kinetics of crystallization. Both models can be used to derive the temperature dependence of the activation energy. The growth model (Equation (13)) gives rise [67] to:

(23) $$E=E_{D}-\frac{\Delta H_{m}\exp\left[\frac{\Delta H_{m}\left(T_{m}-T\right)}{RTT_{m}}\right]}{\exp\left[\frac{\Delta H_{m}\left(T_{m}-T\right)}{RTT_{m}}\right]-1}$$

and the nucleation model (Equation (11)) to:

(24) $$E=E_{D}+\Omega \left[\frac{2T}{{\left(T-T_{m}\right)}^{3}}+\frac{1}{{\left(T-T_{m}\right)}^{2}}\right]$$

Note that Equation (24) is mathematically identical with Equation (16) as can be found by replacing (*T* − *T_m_*) with − (*T* − *T_m_*). Either Equation (23) or Equation (24) predicts E to decrease with increasing *T* and they both have the same asymptotes. When *T* is close to *T_m_* (i.e., at small superheatings) *E* tends to +∞, whereas at large superheatings *E* tends to *E_D_*.

It should be emphasized that the curvature of the Kissinger plots can be more prominent than the one seen in Figure 7. This has been found in the melting kinetic studies on poly(ethylene terephthalate) [68] and poly(ε-caprolactone) [69]. The curvature is larger when melting occurs closer to *T_m_*. In such a case, it can be sufficient to evaluate the actual dependence of *E* vs. *T* that can further be used for estimating the parameters of the nucleation (Equation (24)) and nuclei growth (Equation (23)) models via fitting [67,68,69].

Lastly, one should keep in mind that the models of nucleation and nuclei growth are potentially applicable to a wide variety of phase transitions taking place on heating and cooling. Since it has been already explained that the Kissinger method cannot be applied to the processes taking place on cooling, we only mention examples of phase transitions occurring on heating. These include gelation of aqueous solutions of methylcellulose [70], the coil-to-globule transition in aqueous solutions of poly(*N-*isopropylacrylamide) [71], and solid-solid transition in some salts [72]. All these transitions demonstrate the *E* values that decrease with temperature. In addition, the curvature of the Kissinger plots has been large enough to determine the *E* vs. *T* dependence [71,72]. Fitting Equation (24) to this dependence has afforded estimating the magnitude of the free energy barrier (Equation (12)) and its dependence on temperature.

## 7. Conclusions

An overview of the problems associated with the Kissinger method has been presented. The method is not very accurate. There are different kinds of inaccuracies associated with the assumptions made to derive its basic equations. The assumption of the first-order reaction model normally does not give rise to a large error in the activation energy when the process follows another reaction model. However, this error may become exceedingly large when the process is studied under a temperature program other than the linear heating. The assumption that the kinetics is measured on heating is embedded in the final equation of the method. This makes the Kissinger method unsuitable for any data obtained on cooling.

The method yields a single activation energy that is consistent with the assumption of single-step kinetics. This feature of the method is seen as its most essential problem because the majority of the processes it is applied to are not single-step kinetics. The problem has been illustrated by using common applications of the method. The Kissinger plots tend to be almost perfectly linear in the case of the multi-step kinetics, readily revealed by isoconversional methods. It means that the method tends to yield a single activation energy when a process is controlled by more than one energy barrier. For this reason, the Kissinger method is usually incapable of providing adequate insights into the processes kinetics. As shown by comparison, isoconversional methods provide a significantly more insightful alternative.

To finalize we should note that over the years there have been some developments of the Kissinger method. In particular, efforts have been made to obtain an analytical [73] and exact [74] solution to the Kissinger equation, to adjust it to melt crystallization [12] and reversible decompositions [27], and to exploit the nonlinearity of the Kissinger plots [10]. So far, these developments have had little impact on the mainstream usage of the method that boils down to the straightforward application of the original equation and reporting a single value of the activation energy.
